# Dynamic Changes of Pectin Epitopes in Cell Walls during the Development of the Procambium–Cambium Continuum in Poplar

**DOI:** 10.3390/ijms18081716

**Published:** 2017-08-06

**Authors:** Jundi Liu, Jie Hou, Huimin Chen, Keliang Pei, Yi Li, Xin-Qiang He

**Affiliations:** 1 College of Forestry, Gansu Agriculture University, Lanzhou 730070, China; liujd@gsau.edu.cn (J.L.); peikeliang@163.com (K.P.); liyi@gsau.edu.cn (Y.L.); 2 School of Life Sciences, Peking University, Beijing 100871, China; houjie0325@pku.edu.cn; 3 Hefei No. 1 High School, Hefei 230601, China; hmchen01@126.com

**Keywords:** procambium, cambium, pectin, PME, *Populus tomentosa*

## Abstract

The change of pectin epitopes during procambium–cambium continuum development was investigated by immunolocalization in poplar. The monoclonal antibody JIM5 labels homogalacturonan (HGA) with a low degree of esterification, and the monoclonal antibody JIM7 labels HGA with a high degree of methyl-esterification. Arabinan, rather than galactan, and HGA with low degree of esterification were located in the cell walls of procambial, while HGA with a low degree of esterification was located in the tangential walls, and galactan was located in both the tangential and radial walls of procambial, yet nearly no arabinan was located in the tangential walls of the cambial cells. The changes in pectin distribution took place when periclinal divisions appeared within a procambial trace. The distribution difference of pectin epitopes was also present in procambium–cambium derivatives. The arabinan existed in all cell walls of primary xylem, but was absent from the tangential walls of secondary xylem cells. The galactan existed only in mature primary phloem. Furthermore, 19 pectin methylesterases (PMEs) genes were identified by RNA sequencing, six genes presented highly differentially and were supposed to be involved in the cell wall esterification process. The results provide direct evidence of the dynamic changes of pectin epitopes during the development of the procambium–cambium continuum in poplar.

## 1. Introduction

During the colonization of land, vascular plants acquired the ability to mechanically support the plant body and maintain vigorous growth in the atmosphere [1]. The vascular system of most vascular plant species includes the primary and the secondary vascular systems, which differentiate from procambium and cambium, respectively. Procambium and cambium occur in a developmental community [2,3,4,5]. In *Populus deltoides*, the community is further subdivided into procambium, initiating layer, metacambium and cambium according to the anatomical structure [6,7,8].

Cell growth and differentiation are accompanied by quantitative and qualitative changes in the composition of cell wall polysaccharides [9,10,11]. Pectin is a major component of the primary cell walls of plants and encompasses a range of galacturonic acid-rich polysaccharides [12]. Three major pectic polysaccharides, homogalacturonan (HGA), rhamnogalacturonan-I (RG-I) and rhamnogalacturonan-II (RG-II), occur in all primary cell walls [13]. Developments in analytical techniques and the application of anti-pectin probes have begun to place the structural complexity of pectin in cell biological and developmental contexts [14].

The monoclonal antibodies JIM5 and JIM7 are often used to identify the HGA of low and high methyl degrees, respectively [15,16,17]. The monoclonal antibody LM5 is generated to recognize the (1→4)-β-d-galactan which presents in RG-I side chains [18,19,20,21], and LM6 reacts specifically with (1→5)-α-l-arabinan [22,23]. These antibodies have been used in the studies of secondary vascular systems and provided evidence that the walls of phloem and xylem cells differ in their pectin composition even at a very early stage of commitment [24,25]. However, little is known about the changes of pectin polysacharides during the ontogenetic development of the procambium–cambium continuum.

Pectin methylesterases (PMEs) are ubiquitous enzymes in higher plants and at least 66, 59, and 89 gene family members were identified in Arabidopsis (*Arabidopsis thaliana*), rice (*Oryza sativa*), and poplar (*Populus tomentosa*), respectively [26,27,28]. Recently, based on poplars genome-wide, 24 PMEs have been found in poplar [29]. PMEs are important compositions in plant cell-wall material metabolism [30,31,32,33,34]. Methyl pectin enzymes located in the cell walls of poplar play an important role in bundle cell elongation and development [35]. Poplar has many more gene encoding enzymes that glycosylate secondary metabolites, which could point to more diverse defense mechanisms in woody species [36], such as pectate lyases (PL1 family) and RG-II xylosyl transferases (GT77 family) [37]. Most PME genes (*PttPME1*; *PtxtXET16-34*; *POPTR_0001s44250/Potri.001G416800*; *PtGAUT12.1*) have been shown to participate in poplar apical meristem-provascular, tissue-metavascular, an tissue-secondary vascular tissue-development processes [38,39]. Although the PME family proteins are considered to play potentially diverse physiological roles, the specific functions of the individual members of this family largely remain unknown. In this work, we used a panel of monoclonal antibodies JIM5, JIM7, LM5 and LM6 to map the distribution of pectin epitopes in the different development stages of poplar vascular tissue. Furthermore, RNA sequencing was performed to explore the PME genes involved in the changes of pectin epitopes during the procambium–cambium continuum development in poplar.

## 2. Results

### 2.1. Development of the Procambium–Cambium Continuum in P. tomentosa

Examination of serial sections of apex and stem tissues showed that the procambium–cambium meristematic continuum in *P. tomentosa* could be subdived into procambium, initiating layer, metacambium and cambium, similar to *P. deltoids* [5,6].

**Figure 1:**
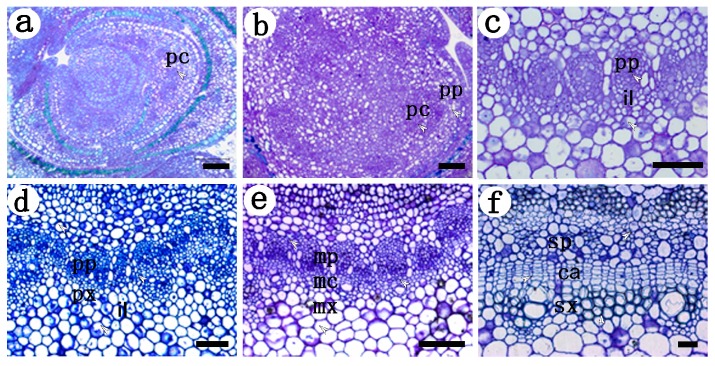
Cross sections of apex and stem at different developmental stages during the procambium–cambium continuum in *P. tomentosa*. (**a**) at the 1 mm level below apex, procambial cells are in the procambium stage, and their divisions were irregular; (**b**) 2 mm below apex, protophloem differentiated on the cortical side of the strand, procambial cells were still in the procambium stage; (**c**) 3 mm below apex, the initiating layer appeared, and the strand was protophloem dominated; (**d**) 8 mm below apex, protoxylem occurred at site internal to the initiating layer; (**e**) 25 mm below apex, the initiating layer had been united into metacambium; (**f**) 200 mm below apex, typical cambium was present. pp Protophloem; px protoxylem; pc procambium; il initiating layer; mx metaxylem; mp metaphloem; mc metacambium; ca cambium; sp secondary phloem; sx secondary xylem. The arrows in the figure indicate the tissues examined. Bar: 10 μm in (**a**,**b**); 20 μm in (**c**–**f**).

The procambium is derived from the residual meristem in the form of acropetally developing strands. The earliest procambial strands could be observed in the transverse sections immediately beneath the apex of *P. tomentosa*. They apparently consist of homogeneous meristematic cells, which are of narrow diameter in the transverse plane and with a dense cytoplasm (Figure 1a). Differentiation with a procambial strand begins while the strand is advancing toward the inception site of its primordium. The first protophloem elements develop on the cortical side of the strand, and then show a centripetal order of differentiation (Figure 1b).

As the strand developed further, some meristematic cells within the strand divided periclinally and formed the first recognizable radially aligned cell, which was the initiating layer (Figure 1c). These periclinal divisions were isolated, so the cells of the initiating layer were tangentially separated from similar radially aligned cells. The initiating layer not only delineated the position of future cambium, but also separate the protophloem tissue from that of the prospective protoxylem. It is notable that the initiating layer did not divide the strand equally and appears well to the inside of the strands. So the strand was phloem-dominated after the initiating layer was established. Later, protoxylem is initiated at a site internal to, but separate from, the initiating layer. It first forms a protoxylem pole and then differentiates centrifugally (Figure 1d).

As additional periclinally dividing cells interposed, the initiating layer united into a tangentially continuous meristem, i.e., metacambium. Metacambium is a more advanced meristematic stage of the continuum, with greater activity of cell division, and more definitive radial and tangential alignment than the initiating layer. The daughter cells produced by the periclinal division of metacambial cells then differentiate into metaphloem and metaxylem (Figure 1e).

Finally, the metacambium grades into cambium at the primary–secondary transition zone. Cambium continually produces daughter cells by periclinal division, which differentiate into secondary phloem outside and xylem inside (Figure 1f).

### 2.2. Distribution of Pectin Epitopes in Procambium–Cambium Continuum Development

#### 2.2.1. Identification of Low Methyl HGA by Monoclonal Antibody JIM5

**Figure 2:**
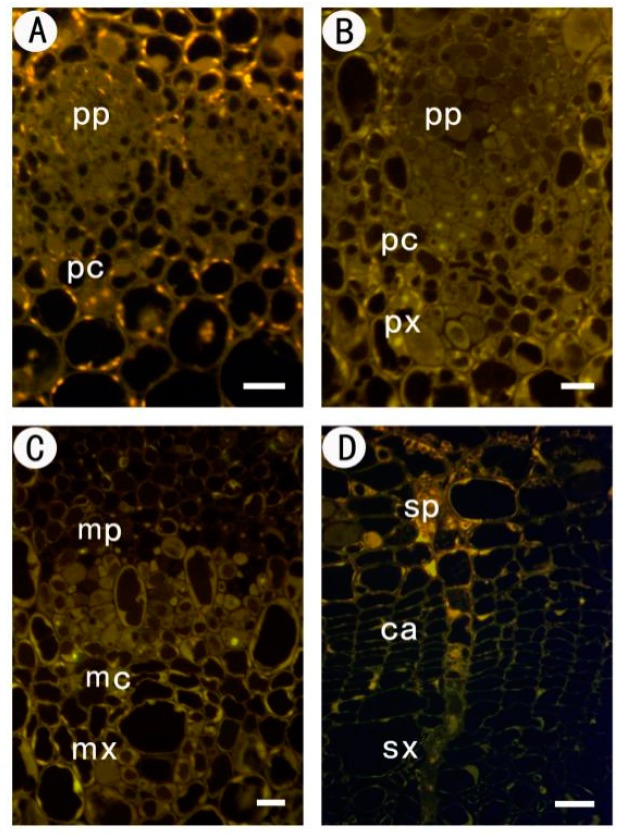
Immunofluorescent labeling without the primary mABs in partial sections at different stages of the procambium–cambium continuum. (**A**) 1 mm level below apex; (**B**) 2 mm below apex; (**C**) 20 mm below apex; (**D**) 200 mm below apex. pp protophloem; px protoxylem; pc procambium; mx metaxylem; mp metaphloem; mc metacambium; ca cambium; sp secondary phloem; sx secondary xylem. Bar: 10 μm in (**A**–**C**); 20 μm in (**D**).

No specific label was detected, but autofluorescence was observed when the primary mABs were omitted from control sections (Figure 2).

**Figure 3:**
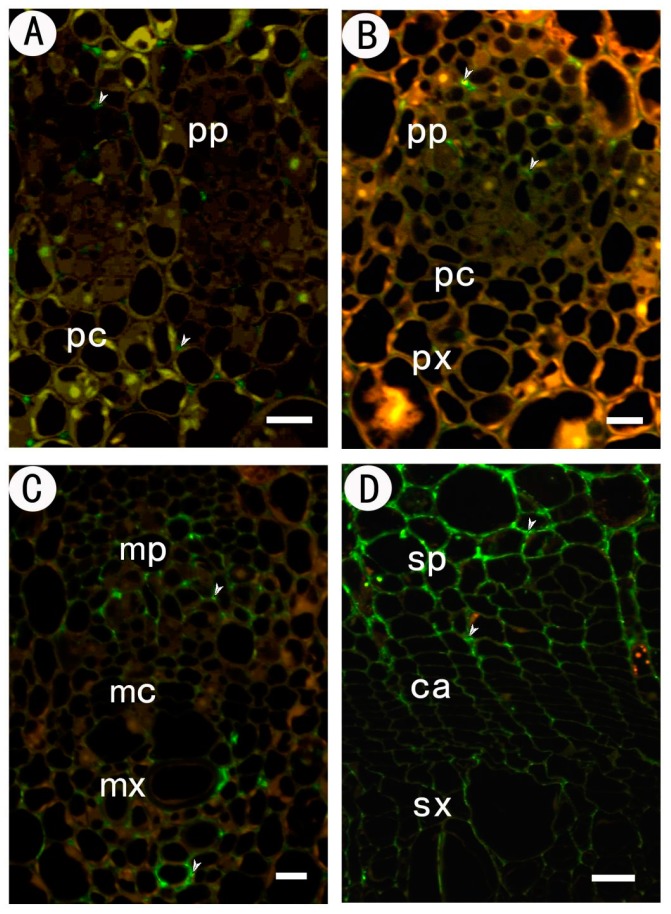
Immunofluorescent labeling with JIM5 antibodies in partial sections at different stages of the procambium–cambium continuum. (**A**) 1 mm level below apex; (**B**) 2 mm below apex; (**C**) 20 mm below apex; (**D**) 200 mm below apex. The arrows in the figure indicate the signal detected. pp protophloem; px protoxylem; pc procambium; mx metaxylem; mp metaphloem; mc metacambium; ca cambium; sp secondary phloem; sx secondary xylem. Bar: 10 μm in (**A**–**C**); 20 μm in (**D**).

The procambium at 1 mm below the stem apex is rarely marked by JIM5. No JIM5 signal was found in the cell walls of the procambium, and only a few signals were able to be detected at the protophloem cells corner (Figure 3A).

The primary vascular tissue at 2 mm below the stem apex had more JIM5 signals at the phloem cells corner. JIM5 signals could also be detected at the old primary xylem cells corner (Figure 3B). Primary vascular tissue was further developed at 20 mm from the stem apex, JIM5 signals could be detected in more cells including newly formed primary phloem and the corners of the primary xylem. At the old primary phloem and the primary xylem, there were not only strong signals at the corners, but also obvious signals at the radial and tangential cell walls. JIM5 signals also appeared at the corners of radial procambium cells (Figure 3C). In the secondary vascular tissues about 200 mm from the stem apex, strong JIM5 signals could be detected in the cell walls of the secondary phloem, especially at the corners. There were nearly no JIM5 signals in the new tangential walls coming from the periclinal division in the cambium region. On the contrary, JIM5 signals were detected in the majority of the radial walls. For the xylem side, as the secondary wall was thickening, JIM5 signals in the primary wall weakened significantly. In the newly formed xylem cells without thickening secondary cell walls, JIM5 signals were merely detected in the tangential cell walls and part of radial cell walls, and there were only JIM5 signals in the corners between cells (Figure 3D).

#### 2.2.2. Identification of High Methyl HGA by Monoclonal Antibody JIM7

**Figure 4:**
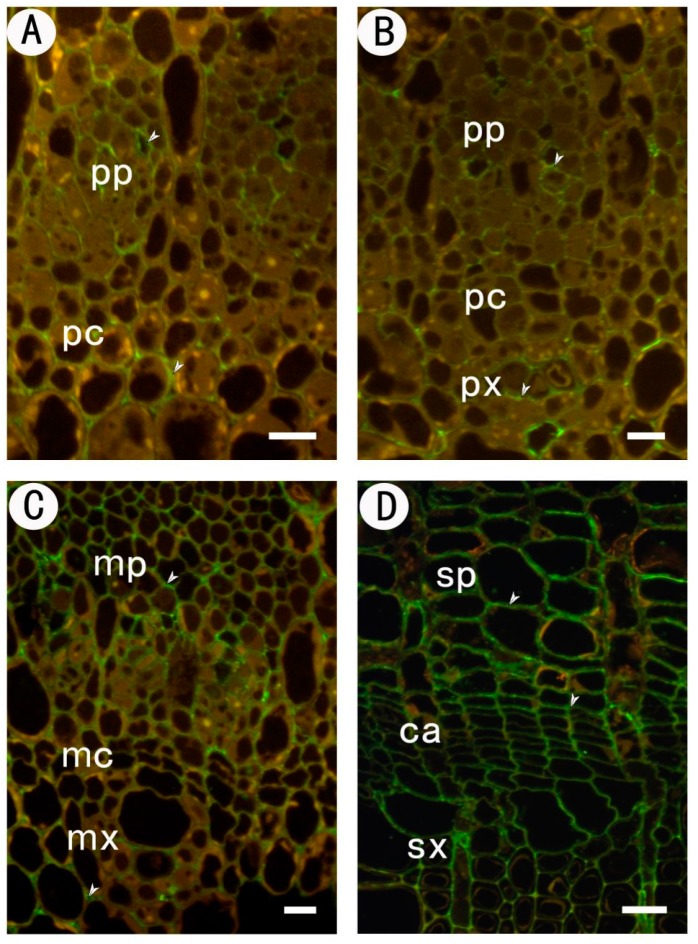
Immunofluorescent labeling with JIM7 antibodies in partial sections at different stages of the procambium–cambium continuum. (**A**) 1 mm level below apex; (**B**) 2 mm below apex; (**C**) 20 mm below apex; (**D**) 200 mm below apex. The arrows in the figure indicate the signal detected. pp protophloem; px protoxylem; pc procambium; mx metaxylem; mp metaphloem; mc metacambium; ca cambium; sp secondary phloem; sx secondary xylem. Bar: 10 μm in (**A**–**C**); 20 μm in (**D**).

JIM7 signals could be detected in cell walls in primary vascular tissues and secondary vascular tissues (Figure 4).

#### 2.3.3. Identification of (1→4)-β-d-galactan in Rhamnogalacturonan-I Side Chains by LM5

**Figure 5:**
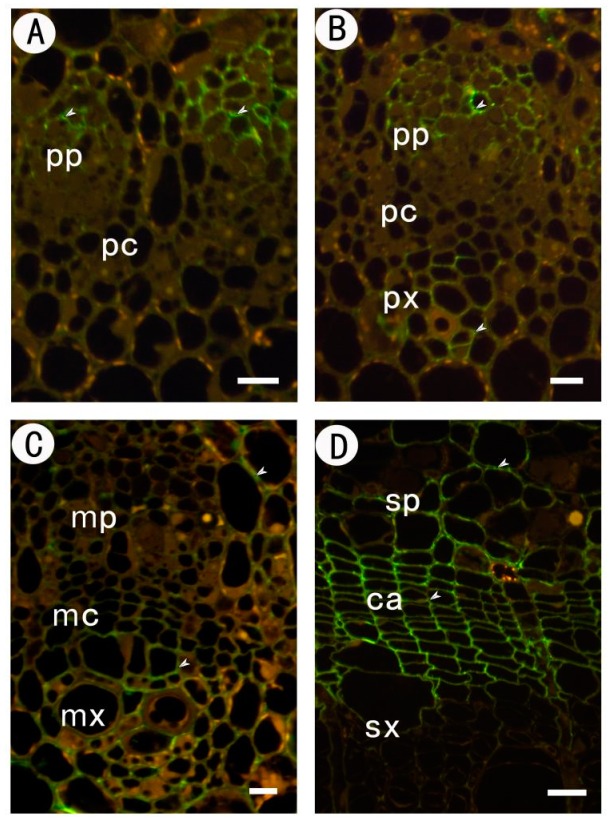
Immunofluorescent labeling with LM5 antibodies in partial section at different stages of the procambium–cambium continuum. (**A**) At the 1 mm level below apex, the cell walls of procambium were not labeled with LM5; (**B**) 2 mm below apex, it was faintly labeled; (**C**) 20 mm below apex; (**D**) 200 mm below apex, cambium and new-formed secondary xylem were intensively labeled with LM5, while secondary phloem was not. The arrows in the figure indicate the signal detected. pp protophloem; px protoxylem; pc procambium; mx metaxylem; mp metaphloem; mc metacambium; ca cambium; sp secondary phloem; sx secondary xylem. Bar: 10 μm in (**A**–**C**); 20 μm in (**D**).

Immunolocalization of (1→4)-β-d-galactan in the procambium–cambium continuum and the LM5 antibody are illustrated in Figure 4. In the early developmental stage when the strands consist of homogeneous procambial cells, no cell wall reacts with LM5 (Figure 5A). Although the cell wall of the procambium and the very young protophloem inside the strand still showed no immunofluorescent reaction, the old protophloem cell wall conspicuously reacted with LM5. Therefore, the LM5 staining showed an increase from the inner to the outer part of the strand.

In the initiating layer stage, the LM5 labelling intensity in the protophloem still increased centrifugally in parallel with cell wall maturation. Weak staining was observed in the cells of the procambium (Figure 5B). Later, the xylem pole appeared and was immediately labelled by LM5. The labelling in xylem cells is intense, whether the cells are young or old. In the metacambium stage, the cell walls of the metacambium were conspicuously labeled with LM5 (Figure 5C). Metaxylem differentiated from metacambium also reacted with LM5, while the metaphloem was devoid of labelling. The labelling pattern in protophloem and protoxylem remained unchanged.

In the cambium stage, intense LM5 labelling was present in the cambial zone (Figure 5D). The labelling vanished in the secondary phloem, while on the secondary xylem side, the labelling remained until the xylem cell wall was secondary-thickened.

#### 2.3.4. Identification of (1→5)-a-l-arabinan in Rhamnogalacturonan-I Side Chains by LM6

**Figure 6:**
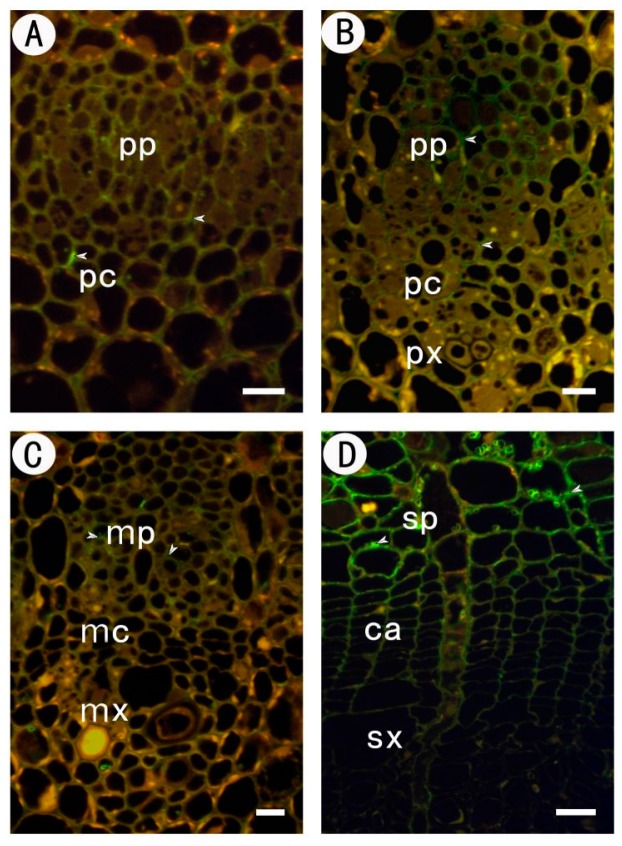
Immunofluorescent labeling with LM6 antibodies in partial sections at different stages of the procambium–cambium continuum. (**A**) At the 1 mm level below apex, the cell walls of procambium were labeled with LM6; (**B**) 2 mm below apex, protophloem and protoxylem were labeled with LM6, while the nascent tangential walls produced by periclinal divisions in the initiating layer were not labeled; (**C**) 20 mm below apex, the tangential walls of metacambium and metaxylem were unlabeled by LM6, while the labelling intensity increased in metaphloem; (**D**) 200 mm below apex, only the radial wall of cambium was faintly labeled. The labelling intensity increased in the secondary phloem side, but decreased in the secondary xylem side. The arrows in the figure indicate the signal detected. pp protophloem; px protoxylem; pc procambium; mx metaxylem; mp metaphloem; mc metacambium; ca cambium; sp secondary phloem; sx secondary xylem. Bar 10 μm in (**A**–**C**); 20 μm in (**D**).

Immunolocalization of (1→5)-α-l-arabinan in the procambium–cambium continuum with the LM6 antibody was shown in Figure 6. In the procambium stage, LM6 labelling was present in all cell walls of the procambium and protophloem (Figure 6A). In the initiating layer stage, the cell walls of procambium, protophloem and protoxylem were still reactive, except the nascent tangential walls produced by periclinal divisions in the initiating layer (Figure 6B). There was no labelling in the tangential walls of the metacambium cells and metaxylem cells, while labelling was detected in metaphloem cells (Figure 6C). A similar labelling pattern was observed in the cambium stage. In this stage, the tangential wall of cambial cells was conspicuously devoid of label, and only faint staining could be seen in the radial wall. The labelling intensity increased in cells committed to the secondary phloem pathway, but decreased in the secondary xylem side (Figure 6D).

#### 2.3.5. PME Gene Family Sequence Analysis and Expression in Procambium–Cambium Continuum Development

**Figure 7:**
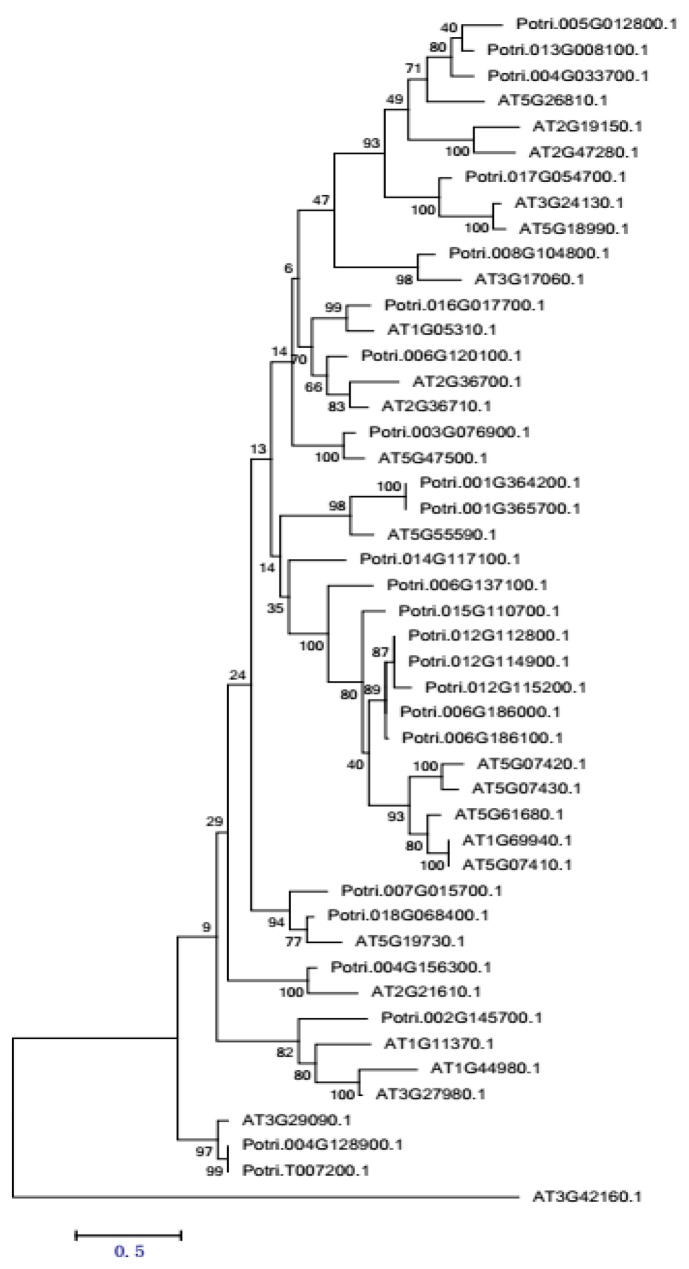
A N-J phylogenetic tree was constructed according to the PME genes sequence similarity of Arabidopsis and *P. trichocarpa*. The phylogenetic tree was built using the biology software MEGA version 6, adopting genetic distance building method (neighbor-joining, NJ), and processing the confidence analysis based on bootstrap by re-sampling 1000 times [40]. The scale (0.5) in the figure presents the bootstrapt value.

PME is a ubiquitous enzyme in higher plants. A total of 23 PMEs were found in *Arabidopsis thaliana*, *Oryza sativa*, *Sorghum bicolor* and *Carica papaya*; 21 in *Vitis vinifera*; 24 in *Populus trichocarpa*; 28 in *Solanum lycopersicum*; 35 in *Physcomitrella patens*; and 18 in *Selaginella moellendorffii*, based on genome-wide PME coding domain recognition [16]. From the phylogenetic tree, *P. trichocarpa* has the highest sequence homology with *A. thaliana*. The PME gene sequences were retrieved from TAIR (http://www.arabidopsis.org/) and Phytozome (http://www.phytozome.net/), respectively. The biology software was employed to construct a phylogenetic tree between PME gene families in *A. thaliana* and *P. trichocarpa* (Figure 7).

**Figure 8:**
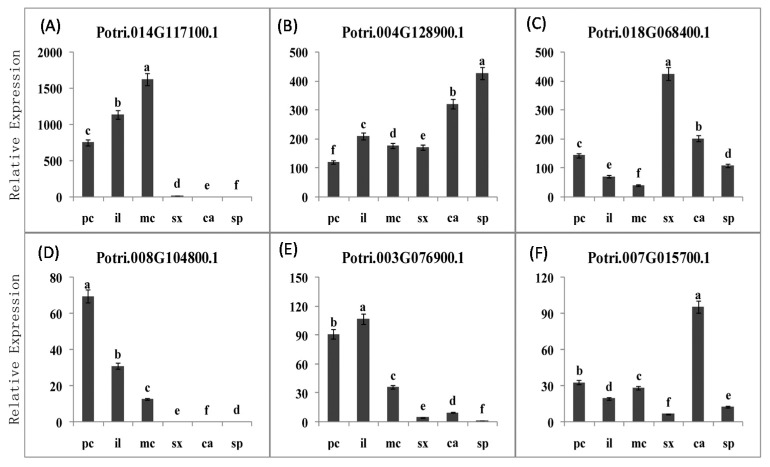
The expression pattern of highly differentially expressed PMEs genes (**A**) Potri.014G117100.1; (**B**) Potri.004G128900.1; (**C**) Potri.018G068400.1; (**D**) Potri.008G104800.1; (**E**) Potri.003G076900.1; (**F**) Potri.007G015700.1) in poplar procambium–cambium continuum development. pc procambium; il initiating layer; mc metacambium; ca cambium; sp secondary phloem; sx secondary xylem.

To further analyze the relationship between the different methyl degrees of HGA and the expression of PME gene families, tangential cryo-sectioning was used to separate poplar vascular tissue samples at different development stages. Before every tangential section, we obtained the upper and bottom cross sections of the material to examine the tissues we would cut. Each tangential section was set at 20 microns thick and about 3–4 layers of cells. To obtain genome-wide insights into the transcriptome changes in vascular tissue development, we used high-throughput RNA sequencing to characterize the cDNA of vascular tissues from the six group samples. We obtained 19 differentially expressed PME genes at different stages, 13 in provascular tissue, 12 in the initiating layer, 13 in metavascular tissue, 10 in the secondary xylem, eight in the cambium, and seven in the secondary phloem. Lastly, six highly differentially expressed PME genes were supposed to be involved in the process based upon their expression patterns (Figure 8). The results provided candidate genes that regulate the changes in dynamics of pectin epitopes during the development of the procambium–cambium continuum in poplar.

To further analyze the relationship between the different methyl degree of HGA and the expression of PME genes family, the tangential cryo-sectioning was used to separate poplar vascular tissue samples of different development stages. Before every tangential section, we get the upper and bottom cross sections of the material to examine the tissues we would cut. Each tangential section was set 20 microns thick about 3–4 layers cells. To obtain genome-wide insights on the transcriptome changes in the vascular tissue development, we used high-throughput RNA sequencing to characterize cDNA of vascular tissues from the six group samples. We obtained 19 differentially expressed PME genes at different stages, 13 in provascurlar tissue, 12 in initiating layer, 13 in metavascular tissue, 10 in secondary xylem, 8 in cambium and 7 in secondary phloem. Lastly, six (6) highly differentially expressed PME genes were supposed to be involved in the process based upon their expression patterns (Figure 8). Three PMEs (a, d and e) appear to be more highly expressed in the primary vascular tissue (pc, il and mc) than in the secondary vascular tissue (sx, ca and sp), while the other three (b, c and f) appear to be express everywhere, but have higher expression level in the secondary vascular tissue.The results provided candidate genes that regulate the dynamics changes of pectin epitopes during the development of procambium-cambium continuum in poplar.

## 3. Discussion

Procambium and cambium are the same meristematic tissues at different stages of development. It was a gradual process from procambium to cambium [6,41,42]. Larson (1976) subdivided the procambium–cambium continuum of *P. deltoides* into procambium, initiating layer, metacambium and cambium, based on anatomical observations [43]. By examining serial sections from apex to stem tissues, we observed the four meristematic stages in *P. tomentosa*. The cytological characteristics changed gradually from procambium to cambium.

Our results showed that the degree of methylation of pectin epitopes in primary vascular tissues and secondary vascular tissues are different. The monoclonal antibody JIM5, recognizing low methyl ester pectin, was rare in the primary vascular tissues, but there was an amount of low methyl ester pectin in the secondary vascular tissues. The monoclonal antibody JIM7 with high methyl ester pectin was identified from procambium to cambium. We inferred that the decrease of the degree of methyl ester in secondary vascular tissue was related to the increase in the secondary cell wall and the enhancement of the support. Degree of immunofluorescent labeling with JIM5, JIM7, LM5 and LM6 antibodies in different tissues during procambium–cambium continuum development was shown in Table 1.

The procambial cell walls reacted with the LM6 antibody, which recognized (1→5)-α-l-arabinan, but did not label LM5, which bound to (1→4)-β-d-galactan. After the procambial cells turned into the initiating layer by periclinal division, arabinan did not occur in the nascent tangential walls, while a spot of galactan appeared in the cell wall of the initiating layer. The remodelling of the pectin side chains in the cell wall of meristematic tissue became more obvious when the initiating layer developed into metacambium and cambium. This also indicated that, although the procambium and cambium were a continuum of the same vascular meristem, the characteristics of their cell walls were different. In the primary growth system, such as the root apex of carrot, flax and Arabidopsis, the galactan epitope was observed to appear at certain points during cell differentiation, while the arabinan epitope was most abundant in meristematic cells [44,45,46,47,48].

During the secondary growth of aspen (*Populus tremuloides*), the galactan epitope was present in the meristematic cells of the cambial zone, while the arabinan epitope was scarce [24]. Our results revealed how the distribution of pectic side chains in the meristematic cells of the primary system gradually converts into that of the secondary system. As the remodelling of pectin side chains in the meristematic tissue of the procambium–cambium community does not occur until the initiating layer appears, the protoxylem-secondary xylem continuum and the protophloem-secondary phloem continuum are derived from the meristematic activity of the procambium–cambium continuum. The protoxylem-secondary xylem continuum is always labeled by LM5, while the protophloem-secondary phloem continuum is always labeled by LM6. It suggests that galactan- and arabinan-rich forms of pectin may give rise to specific mechanical properties required of the cell wall matrix surrounding protoxylem-secondary xylem and protophloem-secondary phloem. The occurrence of galactan-rich forms of pectin may mark the acceleration of cell elongation, as in the Arabidopsis seedling root tip [45] or have a role in increasing the firmness of cell walls, as in pea cotyledons [47].

## 4. Materials and Methods

### 4.1. Plant Material

*P. tomentosa* trees with a stem diameter of 30–40 cm, located on the Peking University campus (39°99′ N, 116°30′ E; Beijing, China), were sampled for the experiments. Sampling was performed from 20 April 2017 until 10 May 2017. Thirty-one year old twigs were collected and fixed in formalin-alcohol-acetic acid (FAA) for anatomical observation. Samples for tangential cryo-sectioning and complete RNA isolation were immediately frozen in nitrogen liquid and stored at −80 °C.

### 4.2. Immunofluorescent Labelling

The entire buds and segments from different internodes were fixed with a mixture of 4% paraformaldehyde and 0.2% glutaraldehyde in 0.1 M phosphate buffer at pH 7.2 for 2 h, dehydrated in an ethanol series, and embedded in LR White. Transverse sections were cut at 4 μm on a Leica microtome (Jung RM 2035). For light microscopy, transverse sections were stained with toluidine blue O (TBO) and observed by light microscopy (Zeiss Axioplan, Jena, Germany).

For immunofluorescence light microscopy, transverse semi-thin sections were collected in multi-well glass slides and incubated with 0.05 M Tris-HC1 (TBS) buffer, pH 7.4–7.6, containing 0.1% Tween 20 (TBS-T buffer). After 10 min, the sections were treated with normal goat serum diluted 1/30 in TBS-T, then incubated overnight at 4 °C with the rat monoclonal antibody (JIM 5, JIM7, LM5 and LM6), diluted 1/4 (*v*/*v*) in TBS-T. Sections were washed three times for 10 min in TBS, then treated with goat anti-rat IgG coupled to fluorescein isothiocyanate (FITC), diluted 1/100 in TBS. After rinsing five times in TBS and twice in distilled water, sections were mounted with a mixture (1:1) of PBS (pH 8.5) and glycerol (containing 3% n-propyl gallate). Sections were examined under a Zeiss microscope with epifluorescent illumination using standard filter combinations for FITC. Control experiments were performed by omitting the primary antibody. No specific label was detected but autofluorescence was observed when the primary MABs were omitted from control sections.

### 4.3. Tangential Cryo-Sectioning, RNA Isolation and RNA Sequencing

A series of 20 μm-thick tangential sections were taken for each sample as described by Uggla and Sundberg (2002) [49] with modification. Tissues at different development stages were isolated by tangential cryosectioning at −24 °C with a Leica CM1850 Cryostat (Leica Microsystems Nussloch GmbH, Nussloch, Germany). Cryosections of tissues at different development stages were collected in a 1.5 mL Eppendorf tube and frozen in nitrogen liquid immediately and then stored at −70 °C for RNA isolation.

Total RNA was isolated with the RNeasy Plant Mini kit (Cat#74904, Qiagen, Dusseldorf, Germany). RNA quality was monitored using a NanoPhotometer P330 (IMPLEN, Munich, Germany). Extracted RNA was used for RNA library construction. The sequencing was carried out by the Biodynamics Optical Imaging Center (BIOPIC, Beijing, China) using Illumina HiSeq 2500 with the single-end program.

## 5. Conclusions

The study firstly traced the change of pectin side chains during the development of the primary–secondary vascular system in poplar. The results showed a significant difference in the distribution of the side chain of RG-I from procambium to cambium. The pectin had a high degree of methyl esterification, and there were more (1→5)-a-l-arabinan and nearly no (1→4)-β-d-galactan in RG-I side chains in the cell wall of the procambium, while this was the opposite for cambium. The results provided direct evidence of the dynamic changes of pectin epitopes during the development of the procambium–cambium continuum, and provided the candidate PME genes regulating the process in poplar.

## Figures and Tables

**Table 1:** Degree of immunofluorescent labeling with JIM5, JIM7, LM5 and LM6 antibodies in different tissues during procambium–cambium continuum development.

Tissues	JIM5	JIM7	LM5	LM6
protophloem	++	+	+	+
procambium	−	+	−	+
protoxylem	+	+	+	+
metaphloem	+++	+ +	+	+
metacambium	+	+	+	−
metaxylem	++	+	+	−
secondary phloem	++++	+++	++	+++
cambium	++	+++	+++	++
secondary xylem	+++	+++	++	+

−: no signals detected; +: faint signals only detected at the corners of the cell walls; ++: strong signals detected at the corners of the cells walls; +++: faint signals detected at the radial and tangential cell walls; ++++: obvious signals detected at the radial and tangential cell walls.
